# Prognostic factors for short-term improvement in acute and persistent musculoskeletal pain consulters in primary care

**DOI:** 10.1186/2045-709X-19-27

**Published:** 2011-11-11

**Authors:** Jennifer E Bolton, Hugh C Hurst

**Affiliations:** 1 Anglo-European College of Chiropractic, Parkwood Road, Bournemouth BH5 2DF, UK; 2 Clifton Chiropractic Clinic, 123 Pembroke Road, Clifton BS8 3EU, UK

**Keywords:** Musculoskeletal, back pain, neck pain, primary care, prognosis, improvement, prospective cohort

## Abstract

**Background:**

Given the costs associated with the management of musculoskeletal pain in primary care, predicting the course of these conditions remains a research priority. Much of the research into prognostic indicators however considers musculoskeletal conditions in terms of single pain sites whereas in reality, many patients present with pain in more than one site. The aim of this study was to identify prognostic factors for early improvement in primary care consulters with acute and persistent musculoskeletal conditions across a range of pain sites.

**Methods:**

Consecutive patients with a new episode of musculoskeletal pain completed self-report questionnaires at baseline, and then again at the 4/5^th ^treatment visit, and if they were still consulting, at the 10^th ^visit. The outcome was defined as patient self-report improvement sufficient to make a meaningful difference. Independent predictors of outcome were identified using multivariate regression analyses.

**Results:**

Acute (<7 weeks) patients, on average, had more severe conditions in terms of pain, disability, anxiety and work fear-avoidance behaviour than patients with persistent (≥7 weeks) pain, but were more likely to be better by the 4/5^th ^visit. Several variables at baseline were associated with improvement at the 4/5^th ^visit, but the predictive models were weak and unable to discriminate between patients who were improved and those who were not. In contrast, it was possible to elicit a predictive model for improvement later on at the 10^th ^visit, but only in patients with persistent pain. Being employed, reporting a decline in work fear-avoidance behaviour at the 4/5^th ^visit, and being better by the 4/5^th ^visit, were all independently associated with improvement. This model accounted for 34.3% (p < 0.001) of the variation in observed improvement, and had good discriminative ability (the area under receiver operating characteristic (ROC) curve was 0.80 (95%CI 0.73 to 0.86)) and approximate balance in correctly identifying improved and non-improved cases (79.0% and 68% respectively).

**Conclusions:**

We were unable to identify baseline characteristics that predicted early outcome in musculoskeletal pain patients. However, early self-reported improvement and decline in work fear-avoidance behaviour as predictors of later improvement highlighted the importance of speedy recovery in persistent musculoskeletal pain consulters. Our findings reinforce the elusive nature of baseline predictors, and the need for more emphasis on early changes as prognostic predictors in musculoskeletal conditions.

## Background

Musculoskeletal disorders, including back, leg, neck, shoulder and arm pain, are costly and prevalent conditions most of which are managed in primary care [1]. The impact of these conditions has ramifications not only for the individual, but at a population level for healthcare utilisation and society at large. In spite of this, our biomedical understanding of the majority of these conditions is poor, and treatment effects are modest at best [2]. Moreover, although the consensus once was that acute episodes recovered within six weeks, it is now known that the clinical course of acute back and neck pain is not that simple, and more often than not beset with frequent recurrences and flare-ups [3-5].

It is apparent that musculoskeletal disorders are highly individualised and multidimensional, and that patients differ both in their response to treatment and in their recovery patterns. This presents enormous challenges to researchers using methodologies in the quantitative paradigm. For example, the modest average treatment effects may mask individual differences with some patients responding a great deal, and others hardly at all. This led, almost a decade ago, to calls for the identification of subgroups of patients either with more favourable prognoses or more likely to respond to particular treatments [2,6].

Unfortunately what was described then as the 'holy grail' of back pain research [7] has yet to produce clear answers in terms of either prognostic or treatment modifier factors. Consistent factors identified between exploratory studies remain elusive, and those factors that have been identified tend to explain relatively little of the variance in the outcome suggesting as yet unidentified predictors. Consequently, research into subgroups has faltered at the derivation stage and has yet to move on significantly to validation and impact studies [6,8].

Nevertheless, given the potential of clinical prediction research in improving patient care in musculoskeletal disorders, the pursuit of prognostic factors continues apace, predominantly in low back and neck and/or shoulder pain. Most recently, investigators in this field have distinguished between acute and chronic patients, arguing that predictors may differ given the different prognostic characteristics of these two groups [4,9,10]. Moreover, others have suggested that prognostic indicators may be common across different pain sites, and rather than distinguish between pain regions, predictors should be identified for musculoskeletal conditions in general [1].

The aim of this study therefore was to identify prognostic factors for short-term improvement in a large cohort of patients with acute and persistent musculoskeletal conditions across different pain sites, typical of those managed in primary care by GPs, physiotherapists, osteopaths and chiropractors.

## Methods

Data from new or new episode patients consulting for a range of musculoskeletal conditions at a chiropractic practice in Bristol, UK from November 2001 to December 2009 were collected prospectively. The only exclusion criteria were patients below the age of 17, and those not able to read and understand the questionnaires either because of limited fluency in the English language or being considered too elderly and frail to do so. Patients were asked to complete questionnaires in the reception area prior to their first consultation (baseline), then again in the reception area after their 4^th ^or 5^th ^treatment session and, if they were still consulting, after their 10^th ^visit. The data from these questionnaires were primarily used to inform the clinician and guide his/her decision-making during the course of treatment. However, patients were informed that the information would also be used anonymously for research purposes and that completion of the questionnaires was indicative of their consent. Ethics approval was given by the local ethics committee. Over the period of data collection, there were a number of chiropractors employed in the practice, and patients were treated pragmatically according to individual needs, including advice, spinal manipulation and mobilisation, dry needling and exercise in keeping with current guidelines [11].

### Questionnaires

The baseline questionnaire collected patient information relating to demographics, work status, lifestyle behaviours and attitudes, and clinical characteristics of the presenting complaint. Areas that the patient felt 'most pain' were categorised as: 'low back'; 'leg(s)'; 'neck'; 'shoulder(s)/arm(s)'; 'head' and 'other'. Patients were allowed to mark more than one area. The questionnaire also included the Bournemouth Questionnaire (BQ), a validated outcome measure for use in routine practice settings in back [12] and neck [13] pain patients. The BQ is a multidimensional instrument that can be used as either individual scores on each of seven 11-point numerical rating subscales covering (i) pain, (ii) disability (activities of daily living (ADL)), (iii) disability (social activities), (iv) anxiety, (v) depression, (vi) work, both inside and outside the home, fear-avoidance and (vii) locus of control, or as the total score (maximum 70). Additionally, patients completed a pain diagram. An overlay grid was used to measure the total area of pain anteriorly and posteriorly (each with a maximum score of 52 units). Inappropriate marking of the pain diagram, for example sporadic, non-physiological patterns, pain markings outside the body, and use of additional words or symbols to describe the pain, was recorded by the clinician. At the 4/5^th ^and 10^th ^visits, patients completed the BQ as well as the Patient Global Impression of Change (PGIC) scale [14].

### Prognostic (predictor) variables

*A priori *discussion between clinicians and researchers identified sociodemographic, biopsychosocial and clinical variables likely to be associated with prognosis in musculoskeletal conditions. Potential predictors were defined either as baseline variables, or as change (between visit) variables. Age, total area of body pain marked on the anterior and posterior pain drawings, and BQ scores and change scores were analysed as continuous variables. All other potential predictors recorded at baseline were dichotomised for ease of interpretation and clinical utility. Area of pain was dichotomised using the mean score for the sample (<8/≥8 units); injury/trauma as a perceived cause of the pain (yes/no/don't know) was collapsed by 'don't know' responses recoded to 'no', expectation of change in condition over the next few weeks (recover or improve/stay about the same/get worse) by recoding to recovery (yes/no), and employment status (employed/working in the home/retired/seeking work) by recoding to paid employment (yes/no). All other variables, which did not require recoding, included pain all over (yes/no), a similar complaint in the past (yes/no), medication use (a lot of the time/occasionally or never), satisfaction with current work status (yes/no), expectation of working normally in six months' time (yes/no), smoker (yes/no), alcohol consumption (regularly/never or hardly ever), level of physical activity compared to people of a similar age (more or about the same/less) and general health and well-being (excellent or good/fair or poor).

### Outcome

Outcomes were monitored using BQ total and sub-scale raw change (i.e. baseline minus treatment visit) scores. The outcome (improvement) for the prediction analysis was defined as those patients scoring either 6 (better, and a definite improvement that has made a real and worthwhile difference) or 7 (a great deal better, and a considerable improvement that has made all the difference) on the PGIC. All other patients (1 = no change or worse, 2 = almost the same, hardly any change, 3 = a little better but no noticeable change, 4 = somewhat better but no noticeable change, 5 = moderately better and a slight and noticeable difference) were categorised as reporting no (meaningful) improvement.

### Data analysis

Baseline characteristics were compared between acute (current episode <7 weeks) and persistent (current episode ≥7 weeks) pain patients using the chi^2 ^test and unpaired t test for categorical and continuous variables respectively. Within-patient differences in continuous variables were analysed using the paired t test.

For the prediction analysis, univariate logistic regression was carried out to determine those variables that were significantly associated with improvement at the 4/5^th ^visit and at the 10^th ^visit. The significance level was set at p ≤ 0.10 to avoid excluding potential predictor variables. Significant variables (p ≤ 0.05) independently associated with the outcome were subsequently entered into a forward stepwise multivariate logistic regression model. Variables were checked for redundancy by noting the correlation coefficients (r≥0.8) between variables in the presence of the other variables in the model. The independence of the variables was also assessed by noting the condition index (<20) of each variable calculated by re-running the final model using linear regression. The ability of the final model to discriminate between improvers and non-improvers was calculated from the area under the receiver operating characteristic curve (AUC) using patient self-report improvement as the external criterion. An AUC of 0.5 indicates no discrimination, 0.7 to 0.8 acceptable discrimination, and 0.9 excellent discrimination [15]. The adjusted %R^2 ^was used as the index of the percentage of the variance in the outcome explained by the model. All analyses were conducted using SPSS v17.

## Results

The baseline questionnaire was completed by 2,422 patients with musculoskeletal complaints amenable to chiropractic treatment. Patients reported pain in the following region(s), which were not mutually exclusive: 1,691 (70.0%) back pain, 973 (40.3%) neck pain, 686 (28.4%) shoulder and/or arm pain, 472 (19.6%) leg pain and 210 (8.7%) headache. Of 1,706 back pain patients, 420 (24.6%) reported accompanying leg pain, and 504 (29.6%) also had neck and/or shoulder/arm pain. Of 1,216 patients presenting with neck and/or shoulder/arm pain, 522 (42.9%) had only neck pain and 231 (19.0%) had only shoulder/arm pain. Almost half (555, 45.8%) of patients with neck and/or upper extremity pain also reported pain in the back and/or leg.

The mean age of the sample was 40.8 (SD ± 14.21) years and the gender split was approximately equal (males: 1,184, 48.9%). The majority of the sample was in paid employment (1,899, 79.4%) with 221 (9.2%) retired, 146 (6.1%) working in the home, 63 (2.6%) seeking work and 62 (2.6%) students. The mean times between baseline and the 4/5^th ^visit, and between baseline and the 10^th ^visit, were 16.8 (± 10.49) and 51.6 (± 15.36) days respectively.

### Acute vs. persistent pain

The characteristics of acute (n = 1,335, 56%) and persistent (n = 1,059, 44%) musculoskeletal pain patients are shown in table 1. Although significant, the difference in age between the two groups was small, and there was no difference in gender distribution. Compared with acute patients, the persistent pain group had a lower proportion in paid employment and a lower proportion satisfied with their work status. As expected, a significantly lower proportion of patients with persistent pain expected to make a good recovery, considered themselves as physically active as people of a similar age and rated themselves in overall good health. Additionally, the mean area of the body shaded in pain was higher in patients with persistent pain, and a higher proportion described their pain as widespread. When reporting the area of their pain, more acute patients reported pain in the back. In contrast a higher proportion of persistent pain patients reported neck pain, upper extremity pain, leg pain and headache. Medication use was significantly higher in the persistent pain group. Interestingly, a statistically significantly higher proportion of acute patients described having similar complaints in the past. Baseline scores on the BQ for acute and persistent pain patients are shown in table 2. Patients in the acute group had statistically higher mean levels of pain and disability in ADL and in social activities, anxiety and work fear-avoidance beliefs.

**Table 1:** Baseline characteristics

Characteristic	Acute (<7 weeks) (n = 1,335)	Missing data	Persistent (≥7 weeks) (n = 1,059)	Missing	*p-value
Gender (male)	674 (50.5)	0	498 (47.0)	0	0.092
Age (years)	40.2 (± 13.09)	0	41.4 (± 15.43)	0	0.032
Pain diagram					
Shaded area (mm):	7.0 (± 5.51)	0	8.3 (± 7.30)	0	<0.001
Posterior	1.5 (± 3.58)	0	2.6 (± 4.79)	0	<0.001
Anterior					
Pain diagram: ≥8 mm	484 (36.3)	0	486 (45.9)	0	<0.001
Pain diagram: Inappropriate marking	145 (10.9)	2 (0.15)	193 (18.2)	2 (0.19)	<0.001
Back pain	984 (73.8)	1 (0.08)	685 (64.9)	4 (0.38)	<0.001
Leg pain	228 (17.2)	6 (0.45)	242 (23.0)	5 (0.47)	<0.001
Neck pain	465 (34.9)	2 (0.15)	501 (47.4)	3 (0.28)	<0.001
Headache	71 (5.3)	2 (0.15)	137 (13.0)	3 (0.28)	<0.001
Shoulder/arm pain	318 (23.9)	3 (0.22)	362 (34.2)	1 (0.09)	<0.001
Widespread pain	23 (1.7)	19 (1.4)	89 (8.6)	28 (2.6)	<0.001
Caused by injury/trauma	354 (26.7)	10 (0.75)	250 (23.9)	11 (1.0)	0.112
Past similar complaint	891 (67.4)	13 (0.97)	640 (62.4)	34 (3.2)	0.012
Taking medication	299 (22.7)	17 (1.3)	279 (26.6)	10 (0.94)	0.028
Expectation of recovery	1,048 (79.1)	10 (0.75)	505 (48.1)	10 (0.94)	<0.001
Paid employment	1,108 (83.8)	13 (0.97)	773 (74.2)	17 (1.6)	<0.001
Satisfied with work	1,191 (93.4)	60 (4.5)	846 (86.6)	82 (7.7)	<0.001
Smoker (ever)	700 (52.5)	2 (0.15)	550 (52.0)	2 (0.19)	0.816
Alcohol (regularly)	1,032 (77.5)	3 (0.22)	718 (68.2)	6 (0.57)	<0.001
Physically active	1,136 (85.3)	4 (0.30)	794 (75.4)	6 (0.57)	<0.001
In good general health	1,204 (90.4)	3 (0.22)	866 (81.8)	7 (0.66)	<0.001

Values are numbers (%) for categorical and mean (± SD) for continuous variables. N = number of observations.

* Chi^2 ^test for categorical and unpaired t test for continuous variables.

**Table 2:** Bournemouth Questionnaire (BQ) scores at baseline and change scores at 4/5^th ^visit

**BQ scores:**	**Baseline**					**Change scores at 4/5^th ^visit**				
	
	**Acute** **(n = 1,335)**	**Missing data**	**Persistent** **(n = 1,059)**	**Missing data**	*p-value	**Acute** **(n = 1,335)**	**Missing data**	**Persistent** **(n = 1,059)**	**Missing data**	*p-value
	
Pain	6.0 (2.12)	14	5.5 (2.21)	3	<0.001	3.4 (2.44)	16	2.1 (2.32)	4	<0.001
Disability in activities of daily living	5.6 (2.69)	13	4.5 (2.75)	1	<0.001	3.5 (2.88)	14	1.8 (2.64)	3	<0.001
Disability in social activities	5.3 (3.07)	18	4.0 (3.03)	6	<0.001	3.4 (3.17)	20	1.7 (2.68)	9	<0.001
Anxiety	5.0 (2.78)	8	4.7 (2.90)	5	0.025	2.7 (2.97)	11	1.8 (2.97)	6	<0.001
Depression	3.2 (2.90)	10	3.3 (2.96)	4	0.504	1.7 (2.81)	13	1.3 (2.89)	8	<0.001
Fear-avoidance beliefs	4.7 (3.06)	34	4.4 (3.02)	16	0.007	2.3 (3.23)	38	1.5 (3.03)	21	<0.001
Locus of control	5.0 (2.59)	20	4.9 (2.78)	12	0.689	2.8 (2.97)	28	1.8 (2.86)	16	<0.001
Total score	34.8 (13.69)	60	31.2 (14.45)	29	<0.001	19.7 (14.67)	77	12.0 (13.29)	43	<0.001

Values are means (± SD). * unpaired t test.

All patients completed a follow-up questionnaire at the 4^th^/5^th ^visit. There was a significant (p < 0.001) decline in all seven BQ sub-scales from baseline to follow up in both groups of patients (table 2). The magnitude of this change however, was significantly greater in the acute group for all seven subscales of the BQ. In terms of improvement (i.e. scoring 6 or 7 on the PGIC scale), as expected a significantly (p < 0.001) higher proportion (941, 70.6%) of patients in the acute group considered themselves as better at the 4/5^th ^visit compared to that (468, 44.3%) in the persistent pain group.

### Prognostic variables

Thirty potential predictor variables for improvement at the 4/5^th ^and at the 10^th ^visits were investigated.

Improvement at 4/5^th ^visit

#### Acute patients

In acute patients, being male, taking medication for the pain complaint, being in paid employment, being a smoker and expecting to make a good recovery were all positively associated with improvement (table 3). Similarly, scoring high at baseline on the BQ sub-scales, apart from depression, increased the odds of improvement at the 4/5^th ^visit. In contrast, having a high area marked on the pain drawing and having upper extremity pain reduced the odds of improvement.

**Table 3:** Univariate logistic regression analysis of potential predictors at baseline for improvement at 4/5^th ^visit

	Acute (n = 1,335)		Persistent (n = 1,059)	
	
Predictor variable	Unadjusted OR (95% CI)	p-value	Unadjusted OR (95% CI)	p-value
Gender (male)	**1.31 (1.03 to 1.66)**	**0.025**	0.92 (0.72 to 1.18)	0.514
*Age (higher)	0.99 (0.99 to 1.00)	0.192	1.00 (0.99 to 1.01)	0.955
*Posterior area body pain (higher)	0.99 (0.97 to 1.01)	0.231	0.99 (0.97 to 1.01)	0.213
*Anterior area body pain (higher)	1.02 (0.98 to 1.06)	0.275	0.99 (0.96 to 1.01)	0.360
Marked area of posterior body pain (high)	**0.77 (0.59 to 0.95)**	**0.018**	0.92 (0.72 to 1.17)	0.494
Inappropriate markings	0.99 (0.68 to 1.44)	0.938	0.81 (0.59 to 1.11)	0.195
Back pain	1.16 (0.89 to 1.51)	0.275	0.81 (0.63 to 1.05)	0.113
Leg pain	0.84 (0.62 to 1.14)	0.268	0.96 (0.72 to 1.28)	0.784
Neck pain	0.90 (0.70 to 1.14)	0.374	**1.34 (1.05 to 1.70)**	**0.020**
Headache	0.80 (0.49 to 1.34)	0.409	1.19 (0.83 to 1.71)	0.342
Shoulder and/or arm pain	**0.69 (0.53 to 0.90)**	**0.007**	1.01 (0.78 to 1.30)	0.968
Widespread pain	0.79 (0.33 to 1.87)	0.584	0.87 (0.56 to 1.35)	0.533
Trauma	1.03 (0.79 to 1.35)	0.804	1.07 (0.80 to 1.42)	0.648
Similar complaint in past	0.94 (0.73 to 1.21)	0.632	0.94 (0.73 to 1.22)	0.657
Taking medication	**1.43 (1.06 to 1.92)**	**0.019**	1.03 (0.78 to 1.36)	0.835
Expect to recover	**1.68 (1.27 to 2.23)**	**0.001**	**1.57 (1.23 to 2.01)**	**0.001**
Paid employment	**1.41 (1.04 to 1.93)**	**0.028**	1.00 (0.76 to 1.33)	0.981
Satisfied with work status	0.80 (0.48 to 1.33)	0.391	1.19 (0.82 to 1.73)	0.368
Smoke	**1.28 (1.01 to 1.62)**	**0.042**	1.02 (0.80 to 1.30)	0.882
Alcohol	0.95 (0.72 to 1.26)	0.728	**0.74 (0.57 to 0.96)**	**0.023**
Physically active	1.18 (0.85 to 1.63)	0.326	1.01 (0.76 to 1.34)	0.948
In good health	1.19 (0.81 to 1.76)	0.384	**1.66 (1.19 to 2.30)**	**0.003**
*BQ Pain	**1.11 (1.05 to 1.17)**	**0.001**	1.03 (0.97 to 1.09)	0.340
*BQ Disability	**1.16 (1.11 to 1.21)**	**0.001**	1.03 (0.99 to 1.08)	0.194
*BQ Social disability	**1.14 (1.10 to 1.19)**	**0.001**	1.02 (0.98 to 1.06)	0.407
*BQ Anxiety	**1.07 (1.03 to 1.12)**	**0.001**	1.03 (0.99 to 1.07)	0.197
*BQ Depression	0.99 (0.95 to 1.03)	0.510	1.01 (0.97 to 1.06)	0.494
*BQ WFAB	**1.06 (1.02 to 1.10)**	**0.003**	1.00 (0.96 to 1.04)	0.962
*BQ LOC	**1.06 (1.02 to 1.11)**	**0.010**	0.98 (0.94 to 1.02)	0.324
*BQ Total	**1.02 (1.01 to 1.03)**	**0.001**	1.00 (1.00 to 1.01)	0.334

*Continuous variables

In the subsequent multivariate analysis (table 4) being male, expecting to make a good recovery, being in paid employment and high levels on the BQ disability scales (ADL and social activities) were all independently associated with improvement. Marking a high area on the pain drawing was independently associated with reduced odds of improvement. However, the model only explained a small amount (7.4%) of variance in the outcome and showed little ability (AUC 0.64) to discriminate between improved and non-improved patients.

**Table 4:** Multivariate logistic regression analysis of prognostic predictors at baseline for improvement at 4/5^th ^visit

	Coefficient	OR	95% CI	p-value	Sensitivity; Specificity; Percentage correctly predicted; Area under ROC (95% CI); Adjusted R^2^
Acute N = 1,335					

Gender (male)	0.26	1.30	1.01 to 1.68	0.044	97.2%; 9.6%; 70.8%; 0.64 (0.61 to 0.68); 7.4%
High area marked on pain drawing	0.27	0.76	0.59 to 0.99	0.040	
Expect to recover	0.34	1.40	1.04 to 1.89	0.025	
In paid employment	0.34	1.40	1.00 to 1.95	0.050	
BQ (ADL disability)	0.085	1.09	1.02 to 1.16	0.012	
BQ (Social disability)	0.072	1.08	1.01 to 1.14	0.016	

Persistent N = 1,059					

Neck pain	0.29	1.34	1.04 to 1.73	0.022	37.0%; 74.1%; 57.7%; 0.60 (0.56 to 0.63); 3.9%
Expect to recover	0.42	1.53	1.19 to 1.96	0.001	
In good health	0.51	1.67	1.19 to 2.35	0.003	
Alcohol	0.29	0.75	0.57 to 0.98	0.034	

#### Persistent pain patients

Fewer variables were associated with improvement at the 4/5^th ^visit (table 3). Patients with neck pain were significantly more likely to be improved, as were patients expecting to make a good recovery and those patients who considered themselves to be in good general health. Patients who reported drinking alcohol had reduced odds of improvement.

In the multivariate analysis (table 4), having neck pain, expecting to make a good recovery and being in good general health were independently associated with improvement at the 4/5^th ^visit. Drinking alcohol was independently associated with reduced odds of improvement. However, the final model again explained very little (3.9%) of the variance in observed improvement, and poor discriminative ability (AUC 0.60).

Improvement at 10^th ^visit

At the 10^th ^visit, the number of patients still consulting was significantly reduced. Of the 1,335 acute patients, improvement data at the 10^th ^visit were available on 168 (11.4%) patients, of whom only 25 (14.9%) reported they were not improved. As a result, there was insufficient data for multivariate regression analysis in this group.

#### Persistent pain patients

For patients with persistent pain, of the original group of 1,059, data were available on 185 (17.5%), of whom 76 (41.1%) were not improved at the 10^th ^visit. Table 5 shows the univariate association between baseline variables, BQ change variables and PGIC data at the 4/5^th ^visit, and improvement at the 10^th ^visit. Reporting a similar condition in the past at baseline was negatively associated with improvement. Being in paid employment, being a smoker and higher baseline BQ scores in disability in ADL and social activities, and in work fear-avoidance behaviour, were all associated with increased odds of improvement at the 10^th ^visit. Considering the change variables, the BQ total and sub-scale change scores, and being better at the 4/5^th ^visit, were all associated with improvement at the 10^th ^visit.

**Table 5:** Univariate and multivariate logistic regression analysis of prognostic predictors (baseline and change) for improvement at 10^th ^visit (persistent pain patients only)

Persistent N = 185	Univariate		Multivariate		
Predictor variable:	Unadjusted OR (95% CI)	p-value	Adjusted OR (95% CI)	p-value	Model
Baseline:					
Gender (male)	1.17 (0.65 to 2.10)	0.602			
*Age (higher)	1.00 (0.98 to 1.01)	0.615			
*Posterior area body pain (higher)	0.98 (0.94 to 1.02)	0.320			
*Anterior area body pain (higher)	0.99 (0.93 to 1.05)	0.732			
Marked area of posterior body pain (high)	1.22 (0.68 to 2.19)	0.510			
Inappropriate markings	0.59 (0.29 to 1.23)	0.158			
Back pain	0.94 (0.49 to 1.78)	0.846			
Leg pain	1.14 (0.57 to 2.29)	0.706			
Neck pain	0.74 (0.41 to 1.34)	0.319			
Headache	0.97 (0.41 to 2.32)	0.950			
Shoulder and/or arm pain	1.28 (0.67 to 2.40)	0.435			
Widespread pain	0.77 (0.27 to 2.22)	0.628			
Trauma	1.44 (0.69 to 2.96)	0.330			
Similar complaint in past	**0.56 (0.28 to 1.09)**	**0.085**			
Taking medication	0.59 (0.31 to 1.13)	0.113			
Expect to recover	1.59 (0.87 to 2.88)	0.130			
Paid employment	**2.06 (1.07 to 3.98)**	**0.031**	**2.26 (1.03 to 4.99)**	**0.043**	
Satisfied with work status	0.80 (0.33 to 1.92)	0.613			
Smoke	**1.85 (1.01 to 3.36)**	**0.045**			
Alcohol	0.67 (0.37 to 1.24)	0.207			
Physically active	1.32 (0.66 to 2.63)	0.429			
In good health	1.77 (0.86 to 3.63)	0.121			
*BQ Pain	1.07 (0.93 to 1.24)	0.338			
*BQ Disability	**1.19 (1.05 to 1.34)**	**0.006**			
*BQ Social disability	**1.10 (0.99 to 1.22)**	**0.086**			
*BQ Anxiety	1.02 (0.93 to 1.13)	0.668			
*BQ Depression	0.99 (0.89 to 1.09)	0.784			
*BQ WFAB	**1.14 (1.02 to 1.27)**	**0.017**			
*BQ LOC	1.00 (0.89 to 1.12)	0.973			
*BQ Total	1.02 (0.99 to 1.04)	0.156			
At 4/5^th ^visit:					
*Change BQ Pain	**1.33 (1.13 to 1.56)**	**0.001**			
*Change BQ Disability	**1.37 (1.19 to 1.58)**	**0.001**			
*Change BQ Social disability	**1.19 (1.05 to 1.35)**	**0.005**			
*Change BQ Anxiety	**1.16 (1.05 to 1.29)**	**0.004**			
*Change BQ Depression	**1.16 (1.04 to 1.29)**	**0.008**			
*Change BQ WFAB	**1.30 (1.16 to 1.47)**	**0.001**	**1.26 (1.11 to 1.44)**	**0.001**	
*Change BQ LOC	**1.27 (1.11 to 1.44)**	**0.001**			
*Change BQ Total	**1.07 (1.04 to 1.10)**	**0.001**			
Improved 4/5^th ^visit	**6.24 (3.15 to 12.36)**	**0.001**	**5.42 (2.59 to 11.35)**	**0.001**	Sensitivity; Specificity; Percentage correctly predicted; Area under ROC (95% CI); Adjusted R2 79.0%; 68.0%; 74.4%; 0.80 (0.73 to 0.86); 34.3%

*Continuous variables

To avoid over-fitting the model given the relatively small number of patients (109 outcome events (improvement)), only those predictor variables significant at the 5% level were entered in the multivariate model. The change in total BQ was excluded to avoid colinearity between this and the BQ sub-scale scores. From the 12 variables entered, three variables were independently associated with improvement at the 10^th ^visit (table 5). These were improvement at the 4/5^th ^visit, being in paid employment, and decline in work fear-avoidance behaviour at the 4/5^th ^visit. The model correctly predicted 74.4% of patients (compared to the observed 58.3%) improving at the 10^th ^visit. The predictive model accounted for 34.3% of the variation in observed improvement, had good discriminative ability (AUC 0.80) and an approximate balance of correctly identifying improved and non-improved cases (79.0% and 68% respectively).

## Discussion

This study identified prognostic variables in patients consulting for acute and persistent musculoskeletal pain conditions. We chose to use patient self-report improvement as the outcome, and used a rigorous cut-off for improvement that was meaningful to the patient. It has been shown that patients' perception of improvement is multi-factorial and more complex than either alleviation of symptoms or improvement in function alone [16,17]. We made the distinction between acute and persistent pain in line with the NICE guideline on the management of pain greater than 6 weeks [11]. Our results showed clear differences, on average, between these two groups in terms of more area in pain, widespread pain and lower expectations of recovery in those with persistent pain. In contrast, acute patients were more likely to be in employment, satisfied with their work status, and consider themselves to be physically active and in general good health. Musculoskeletal conditions in acute patients were more severe at baseline, with higher levels of pain, disability, anxiety and work fear-avoidance behaviour than those with persistent pain. In spite of greater severity however, acute patients got better faster, and by the 4/5^th ^visit (approximately 2 weeks on average) a greater proportion reported that they were better.

A significant proportion of patients in this study reported pain in more than one site, illustrating the difficulty of investigating musculoskeletal disorders on the basis of regional pain. In spite of this, most musculoskeletal research in primary care confines itself to single pain sites, for example low back pain, even though inevitably a proportion of participants will have musculoskeletal pain elsewhere. In spite of this, in a systematic review of prognostic factors in musculoskeletal pain, only 4 out of 45 included studies investigated patients with general musculoskeletal pain [1].

Expecting to make a good recovery was strongly associated with early improvement by the 4/5^th ^visit in both acute and persistent pain patients, but apart from this no other predictors were the same in these two groups. This contrasts with the study by Grotle et al. [10] that suggested considerable overlap in prognostic indicators for outcome between acute (<3 months) and chronic (>3 months) low back pain patients. Similar overlap in prognostic factors was found in patients with acute (<2 weeks) [4] and chronic (> 3 months) [9] low back pain. In the present study, being male, being in paid employment, being a smoker, high BQ baseline levels and taking medication were all associated with early recovery in acute patients, while marking a higher area on the pain drawing and having upper extremity pain were negatively associated. In patients with persistent pain, having neck pain and reporting being in good health were positively associated, whereas alcohol consumption was negatively associated. However, in both acute and persistent pain patients the predictive models explained very little of the variance in improvement by 4/5^th ^visit, suggesting that other factors, not measured in this study, are important. For example, longer pain duration has been identified as one of the few consistent predictive factors in back and neck pain [1]. However, as we used this variable to subgroup our population, it was not entered as a potential predictor. Similarly, we did not measure improvement before the 4^th ^visit, which in another prognostic study in chiropractic patients, being better at the 2^nd ^visit was identified as a strong predictor of improvement at the 4^th ^[18]. It may also be the case that it is not possible to predict early recovery from baseline variables, particularly in acute patients with generally rapid recovery.

We were unable to investigate prognostic factors for improvement at the 10^th ^visit in acute patients because most of these patients had ceased to consult. Although this might be presumed to be because they were no longer in pain, this may not be the case [3]. In contrast, sufficient numbers of persistent pain patients still consulting allowed us to proceed with analysis. Being in paid employment, being a smoker, and high levels of baseline disability and work fear-avoidance behaviour were all positively associated with improvement by the 10^th ^visit, while having a similar condition in the past was negatively associated. Declines in all the BQ sub-scales between baseline and the 4/5^th ^visit, and reporting improvement on the PGIC scale at the 4/5^th ^visit, were also positively associated with improvement at the 10^th ^visit. In the final predictive model, being better at the 4/5^th ^visit was by far the strongest predictor, together with being in paid employment and reporting a decline in work fear-avoidance behaviour at the 4/5^th ^visit. This result echoes that shown by others [18,19] that early changes may be more important as predictors in musculoskeletal conditions than variables measured at baseline. In other words, that unless a patient improves quickly, the prognosis, at least in the short-term, is poor. It also calls into question the use of screening tools at baseline to identify patients at risk of poor prognosis, and the need for inclusion of early changes in addition to baseline factors in future prognostic research. The other factors that were independently associated with improvement were a decline in work fear-avoidance behaviour and being in paid employment. In a recent study, fear of pain was found to be a significant predictor of outcome in back pain patients [10], although a systematic review of fear-avoidance behaviour and prognosis in back pain [20] reported no convincing evidence of a link. In support of our findings on employment status as a prognostic factor, Grotle and co-workers [10] showed that being non-employed was associated with long term disability in both acute and chronic low back pain patients. In overall terms, when comparing our findings with the existing literature, it is clear that there remains a lack of consistency in prognostic factors identified between studies, and that identifying useful factors, particularly at baseline, remains a challenge.

This study has several limitations, not least because it was a pragmatic study conducted in real time everyday clinical practice. First, it is restricted to relatively early outcomes (up to seven weeks on average) and in the relatively small proportion of patients that were still consulting. We therefore do not know if the predictive model presented here holds true either in the longer term or in those patients who had stopped consulting. Second, there was no control group, and therefore we are only able to present prognostic factors for the clinical course of these conditions, and not for treatment effects. Third, all variables were self-reported by patients in the chiropractic practice, which may have been subject to reporting bias. Fourth, all patients attended one chiropractic practice, and as such may not be representative either of musculoskeletal patients in general seeking chiropractic care, or of those seeking care from other primary care practitioners. Finally, while it may be considered a strength of the study to include patients with musculoskeletal complaints in general, there still remains the question of why should prognostic factors be common across musculoskeletal pain sites. Many of these conditions share similar characteristics, such as co-morbidities, non-specific aetiologies, recurrent and episodic courses, and prognostic factors such as duration of pain and previous episodes. Nevertheless, clumping these conditions together may be a step too far, or alternatively beg the question as to why not more musculoskeletal research is conducted on this basis.

## Conclusions

Patients with acute musculoskeletal pain consulting a chiropractor were more severe, on average, than those with persistent pain, but more likely to get better quicker. Although a number of variables were associated with a favourable outcome in acute and persistent pain patients by the 4/5^th ^visit, the predictive models were weak and we were unsuccessful in identifying baseline variables as predictors of early recovery. In contrast, the model constructed to predict self-reported improvement later on in persistent musculoskeletal pain patients showed good discriminative ability. In this model, reporting improvement at the 4/5^th ^visit, a decline in work fear-avoidance behaviour at the 4/5^th ^visit and being in paid employment at baseline were all independently associated with improvement at the 10^th ^visit. These results highlight the importance of early recovery in persistent musculoskeletal pain consulters, and the need to include change variables in addition to baseline variables in future prognostic research.

## Competing interests

The authors declare that they have no competing interests.

## Authors' contributions

JB and HH conceived and designed the study; HH collected the data; JB and HH analysed and interpreted the data. JB was responsible for the first draft of the manuscript; JB and HH were responsible for revisions and approval of the final manuscript.
